# Robust hypergraph regularized non-negative matrix factorization for sample clustering and feature selection in multi-view gene expression data

**DOI:** 10.1186/s40246-019-0222-6

**Published:** 2019-10-22

**Authors:** Na Yu, Ying-Lian Gao, Jin-Xing Liu, Juan Wang, Junliang Shang

**Affiliations:** 1 School of Information Science and Engineering, Qufu Normal University, Rizhao, 276826 China; 2 Library of Qufu Normal University, Qufu Normal University, Rizhao, 276826 China

**Keywords:** Non-negative matrix decomposition, Hypergraph Laplacian, L_2,1_-norm, Clustering, Common abnormal gene selection, Multi-view gene expression data

## Abstract

**Background:**

As one of the most popular data representation methods, non-negative matrix decomposition (NMF) has been widely concerned in the tasks of clustering and feature selection. However, most of the previously proposed NMF-based methods do not adequately explore the hidden geometrical structure in the data. At the same time, noise and outliers are inevitably present in the data.

**Results:**

To alleviate these problems, we present a novel NMF framework named robust hypergraph regularized non-negative matrix factorization (RHNMF). In particular, the hypergraph Laplacian regularization is imposed to capture the geometric information of original data. Unlike graph Laplacian regularization which captures the relationship between pairwise sample points, it captures the high-order relationship among more sample points. Moreover, the robustness of the RHNMF is enhanced by using the L_2,1_-norm constraint when estimating the residual. This is because the L_2,1_-norm is insensitive to noise and outliers.

**Conclusions:**

Clustering and common abnormal expression gene (com-abnormal expression gene) selection are conducted to test the validity of the RHNMF model. Extensive experimental results on multi-view datasets reveal that our proposed model outperforms other state-of-the-art methods.

## Background

Due to the development of sequencing technology, more and more gene expression data have been detected. At the same time, there are many meaningful biological information in gene expression data. The effective analysis and research of this information are of great significance to the prevention and treatment of diseases. And multi-view data obtained by integrating data from different sources have gained much attention in the field of machine learning [1]. It is well known that gene expression data can be downloaded from The Cancer Genome Atlas (TCGA) platform. We then integrated the gene expression data into multi-view data for different diseases with the same genes. Multi-view data will provide a new perspective to mine the connections between multiple cancers.

To meet the demand for studying explosive gene expression data, modern biologists are increasingly concerned with clustering and feature selection. Clustering is the process of dividing a series of genes or samples into different subsets, and the genes or samples in the same subset are similar [2]. Generally speaking, feature selection can not only find useful information and eliminate noise, but also reduce the complexity of the computation. In this paper, we performed the selection of com-abnormal genes to study the relationship between genes and multiple cancers [3].

As an effective matrix decomposition method, non-negative matrix factorization (NMF) [4] is widely prevalent in bioinformatics [5], image representation [6], and other fields [7]. NMF can learn part-based representations of objects. This is consistent with the human brain’s perception mechanism. Some extensions to NMF have been proposed from different perspectives. For example, the non-negative local coordinate factorization (NLCF) was presented by imposing the locality coordinate constraint into the original NMF [8]. Kim et al. presented the sparse non-negative matrix factorization (NMFs) method in combination with sparse constraints [9]. In practical applications, the data are sometimes negative, so semi-non-negative matrix factorization (Semi-NMF) and convex non-negative matrix factorization (Convex-NMF) are derived to solve the problem of positive and negative data [10].

As we mentioned above, these methods have enhanced the performance of NMF, but there also exist the following limitations: (1) In fact, there is an intrinsic geometrical information in the high-dimensional data. But these methods ignore the nonlinear low-dimensional geometrical structure in the original data. (2) There are always noise and outliers in real data. Therefore, we need a robust NMF-based approach to effectively suppress noise and outliers.

For the first question, the graph regularized non-negative matrix factorization (GNMF) was presented to discover the manifold structure of raw data [11]. However, graph regularization is based on constructing k-nearest neighbors in a simple graph, which explores only the pairwise relationship between two sample points. Zeng et al. introduced hypergraph regularized non-negative matrix factorization (HNMF) to encode the relationship between two or more than two sample points [12]. Unlike simple graphs, the hyperedge of a hypergraph contains a series of related vertices. Therefore, high-order relationship of the data can be found. GNMF and HNMF consider important manifold information, but they are exceptionally sensitive to noise and outliers. For the second problem, using the *L*_2, 1_-norm when estimating the residual can be effectively alleviated [13].

Inspired by these work, this paper presents a novel NMF model called robust hypergraph regularized non-negative matrix factorization (RHNMF). It adds hypergraph regularization and *L*_2, 1_-norm to the traditional NMF. So it has the advantage of considering the higher-order relationship among samples and controlling the influences of noise and outliers. The main contributions of RHNMF are summarized as follows:

(i) To capture high-order relationship between more sample points, hypergraph regularization is applied to the objective function. This makes sense for enhancing the performance of NMF-based methods.
(ii) The *L*_2, 1_-norm instead of the Frobenius norm is used to estimate the residual approximation, so that the error term for each data point is no longer squared form. This will greatly suppress the effects of noise and outliers. And *L*_2, 1_-norm is suitable for clustering and feature selection because it produces sparse rows.
(iii) Scientific and comprehensive experiments are designed on the multi-view datasets to prove the effectiveness of the RHNMF and achieved satisfactory results.

The rest of the paper is arranged as follows. In the “Methods” section, we introduce the NMF, *L*_2, 1_-norm, and hypergraph regularization. The proposed RHNMF method, the solution process, its convergence, and computational complexity analysis are also described in detail. Experimental results are demonstrated in the section “Results and discussion.” The conclusion is drawn in the section “Conclusions.”

## Methods

### Non-negative matrix factorization

In the field of bioinformatics, gene expression data are usually expressed in the form of a matrix. The sample is represented by a column of matrices, and the level of gene expression is represented by the rows of the matrix. Given a data matrix **X** = [**x**_1_, **x**_2_, …, **x**_*n*_] ∈ *R*^*m* × *n*^, the column vector **x**_*j*_ is a sample vector. NMF aims at finding two non-negative matrices **U** = [**u**_1_, **u**_2_, …, **u**_*k*_] ∈ *R*^*m* × *k*^ and **V** = [**v**_1_, **v**_2_, …, **v**_*n*_] ∈ *R*^*k* × *n*^ whose products are similar to the data matrix **X** [14]. **U** represents a basis matrix, and **V** represents a coefficient matrix. The minimizing objective function of the NMF is as below:

1 $$ \underset{\mathbf{U},\mathbf{V}}{\min }{\left\Vert \mathbf{X}-\mathbf{UV}\right\Vert}_F^2=\sum \limits_{j=1}^n{\left\Vert {\mathbf{x}}_j-{\mathbf{U}\mathbf{v}}_j\right\Vert}^2\kern0.5em s.t.\mathbf{U}\ge 0,\mathbf{V}\ge \mathbf{0},\kern0.5em $$

where ‖⋅‖_*F*_ represents the Frobenius norm of matrix. **x**_*j*_ can be seen as a linear combination of columns of **U**, parameterized by each column of **V** [15].

### *L*_2, 1_-norm

Given any matrix **X** ∈ *R*^*m* × *n*^, the ‖**X**‖_2, 1_ is to first calculate *L*_2_-norm for rows to form a column matrix, and then calculate *L*_1_-norm for column matrix [13], i.e.,

2 $$ {\left\Vert \mathbf{X}\right\Vert}_{2,1}=\sum \limits_{i=1}^m\sqrt{\sum \limits_{j=1}^n{\mathbf{x}}_{i,j}^2}=\sum \limits_{i=1}^m{\left\Vert {\mathbf{x}}^i\right\Vert}_2 $$

As shown above, *L*_2, 1_-norm will cause row sparsity [16]. At the same time, the *L*_2, 1_-norm is not susceptible to noise and outliers, so the robustness of the algorithm can be improved.

### Hypergraph regularization

Inspired by the simple graph theory, hypergraph came into being [17]. In the sample graph, one edge is connected by two data samples and the weight of the edge denotes the pairwise relationship between two sample points [11]. To solve this problem, hypergraph takes into account the relationships between multiple vertices and construct hyperedges for them [12].

**Fig. 1:**
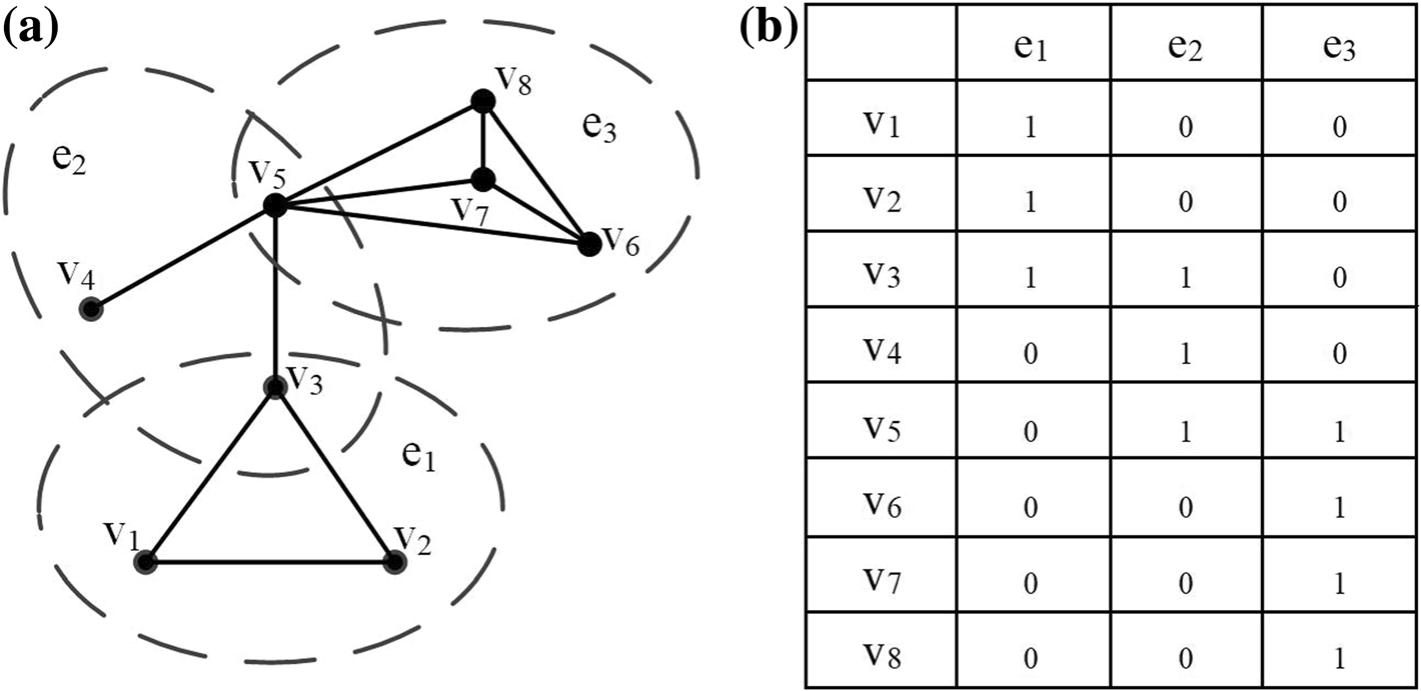
Illustration of the hypergraph. **a** An example of a hypergraph. **b** Its corresponding incidence matrix

Let a triple *G* = (*V*, *E*, **W**) represent a hypergraph, where vertex set is represented by *V*, hyperedge set is *E*, and **W** is the diagonal matrix that represents the weights of the hyperedges. As shown in Fig. 1a, this is an example of a hypergraph. There are six vertices and three hyperedges in this hypergraph. Then, the hyperedge set *E* = {*e*_1_ = {*v*_1_, *v*_2_, *v*_3_}, *e*_2_ = {*v*_3_, *v*_4_, *v*_5_}, *e*_3_ = {*v*_5_, *v*_6_, *v*_7_,*v*_8_}}. In Fig. 1b, **H** ∈ *R*^|*V*| × |*E*|^ represents the hypergraph’s incidence matrix. Then, we can calculate it as below:

3 $$ \mathbf{H}\left(v,e\right)=\left\{\begin{array}{c}1\kern2.2em if\kern0.1em v\in e,\\ {}0\kern1.25em \mathrm{otherwise}.\end{array}\right. $$

For any hyperedge *e*_*i*_, its weight **W**_*i*_ is denoted as follows:

4 $$ {\mathbf{W}}_i=\mathbf{W}\left({e}_i\right)=\sum \limits_{v_j\in {e}_i}\exp \left(-\frac{{\left\Vert {v}_i-{v}_j\right\Vert}_2^2}{\delta}\right), $$

where $$ \delta =\raisebox{1ex}{$1$}\!\left/ \!\raisebox{-1ex}{$k$}\right.\sum \limits_{v_j\in {e}_i}{\left\Vert {v}_i-{v}_j\right\Vert}_2^2 $$, *k* represents the value of *k* nearest neighbors for each vertex. *d*(*v*) represents the degree of vertex *v* and is expressed as follows:

5 $$ d(v)=\sum \limits_{e\in E}w(e)\mathbf{H}\left(v,e\right). $$

And the degree of each hyperedge can be denoted as:

6 $$ f(e)=\sum \limits_{v\in V}\mathbf{H}\left(v,e\right). $$

The unnormalized hypergraph Laplacian matrix [17] is defined as:

7 $$ {\mathbf{L}}_{\mathrm{hyper}}={\mathbf{D}}_v-\mathbf{E}, $$

where **E** = **HW**(**D**_*e*_)^−1^**H**^*T*^ and **D**_*v*_ is a diagonal matrix composed of *d*(*v*). **W** denotes a diagonal matrix composed of *w*(*e*). **D**_*e*_ used to represent the diagonal matrix composed of *f*(*e*).

Hypergraph regularization [12] can be defined to minimize the following optimization problem:

8 $$ {\displaystyle \begin{array}{l}\kern0.5em \underset{\mathbf{V}}{\min}\frac{1}{2}\sum \limits_{e\in E}\sum \limits_{\left(i,j\right)\in e}\frac{w(e)}{f(e)}{\left\Vert {\mathbf{s}}_i-{\mathbf{s}}_j\right\Vert}^2\\ {}=\underset{\mathbf{V}}{\min } Tr\left(\mathbf{V}\left({\mathbf{D}}_v-\mathbf{E}\right){\mathbf{V}}^T\right)\\ {}=\underset{\mathbf{V}}{\min } Tr\left({\mathbf{V}\mathbf{L}}_{\mathrm{hyper}}{\mathbf{V}}^T\right),\end{array}} $$

where **s**_*i*_ and **s**_*j*_ are low-dimensional representations of the original data points **x**_*i*_ and **x**_*j*_.

### The proposed method: robust hypergraph regularized non-negative matrix factorization (RHNMF)

Traditional NMF is a good part-based representation algorithm [4]. However, its objective function is a form of square residual. Therefore, traditional NMF is susceptible to noise and outliers. Moreover, NMF ignores the low-dimensional manifold embedded in the high-dimensional data.

To overcome the above limitations, we present a new method called RHNMF. It considers the robustness of the algorithm and the high-order relationship between the data. In other words, RHNMF method is the integration of NMF, *L*_2, 1_-norm, and hypergraph. The objective function of RHNMF is defined as follows:

9 $$ \underset{\mathbf{U},\mathbf{V}}{\min }{\left\Vert \mathbf{X}-\mathbf{UV}\right\Vert}_{2,1}+\alpha Tr\left({\mathbf{V}\mathbf{L}}_{\mathrm{hyper}}{\mathbf{V}}^T\right)\kern1em s.t.\mathbf{U}\ge 0,\mathbf{V}\ge 0, $$

where *Tr*(⋅) represents the trace of the matrix and *α* ≥ 0 denotes the weighting parameter to balance two terms.

### Solution of RHNMF

By using ‖**B**‖_2, 1_ = *Tr*(**BDB**^*T*^), the objective function in Eq. (9) is expressed as follows:

10 $$ {\displaystyle \begin{array}{l}\kern0.5em Tr\left(\left(\mathbf{X}-\mathbf{UV}\right)\mathbf{D}{\left(\mathbf{X}-\mathbf{UV}\right)}^T\right)+\alpha Tr\left({\mathbf{V}\mathbf{L}}_{\mathrm{hyper}}\mathbf{V}\right)\\ {}= Tr\left({\mathbf{XDX}}^T\right)-2 Tr\left({\mathbf{XDV}}^T{\mathbf{U}}^T\right)+ Tr\left({\mathbf{U}\mathbf{V}\mathbf{DV}}^T{\mathbf{U}}^T\right)\\ {}\kern0.6em +\alpha Tr\left({\mathbf{V}\mathbf{L}}_{\mathrm{hyper}}{\mathbf{V}}^T\right),\end{array}} $$

where **D** denotes the diagonal matrix with *i*th diagonal element as

11 $$ {\displaystyle \begin{array}{c}{\mathbf{D}}_{jj}=\raisebox{1ex}{$1$}\!\left/ \!\raisebox{-1ex}{$\sqrt{\sum_{m=1}^i{\left(\mathbf{X}-\mathbf{U}\mathbf{V}\right)}_{m,j}^2+\gamma }$}\right.\\ {}\kern1.5em =\raisebox{1ex}{$1$}\!\left/ \!\raisebox{-1ex}{$\left\Vert {x}_j-\mathbf{U}{v}_j+\gamma \right\Vert $}\right.,\end{array}} $$

where *γ* represents the sufficiently small positive number infinitely close to 0 but not 0. The multiplication update rule is used to iteratively update the objective function to minimize the error. Then, the Lagrangian function *f* can be expressed as

12 $$ {\displaystyle \begin{array}{l}f= Tr\left({\mathbf{XDX}}^T\right)-2 Tr\left({\mathbf{XDV}}^T{\mathbf{U}}^T\right)+ Tr\left({\mathbf{U}\mathbf{VDV}}^T{\mathbf{U}}^T\right)\\ {}\kern1.3em +\alpha Tr\left({\mathbf{V}\mathbf{L}}_{\mathrm{hyper}}{\mathbf{V}}^T\right)+ Tr\left({\boldsymbol{\uppsi} \mathbf{U}}^T\right)+ Tr\left({\boldsymbol{\upvarphi} \mathbf{V}}^T\right),\end{array}} $$

where **ψ** = [*ψ*_*ik*_] and **φ** = [*φ*_*kj*_] denote Lagrange multipliers which are constrained to **U** ≥ **0** and **V** ≥ **0**, respectively.

The partial derivative of *f* with respect to **U** and **V** can be defined as follows:

13 $$ \frac{\partial f}{\partial \mathbf{U}}=-2{\mathbf{XDV}}^T+2{\mathbf{UVDV}}^T+\boldsymbol{\uppsi}, $$

14 $$ \frac{\partial f}{\partial \mathbf{V}}=-2{\mathbf{U}}^T\mathbf{XD}+2{\mathbf{U}}^T\mathbf{UVD}+2\alpha {\mathbf{VL}}_{\mathrm{hyper}}+\boldsymbol{\upvarphi} . $$

The iterative formulas of the objective function are expressed as follows:

15 $$ {u}_{ik}\leftarrow {u}_{ik}\frac{{\left({\mathbf{XDV}}^T\right)}_{ik}}{{\left({\mathbf{UVDV}}^T\right)}_{ik}}, $$

16 $$ {v}_{kj}\leftarrow {v}_{kj}\frac{{\left({\mathbf{U}}^T\mathbf{XD}+\alpha \mathbf{VE}\right)}_{kj}}{{\left({\mathbf{U}}^T\mathbf{UVD}+\alpha {\mathbf{VD}}_v\right)}_{kj}}. $$

Then, the corresponding algorithm is given in Algorithm 1.
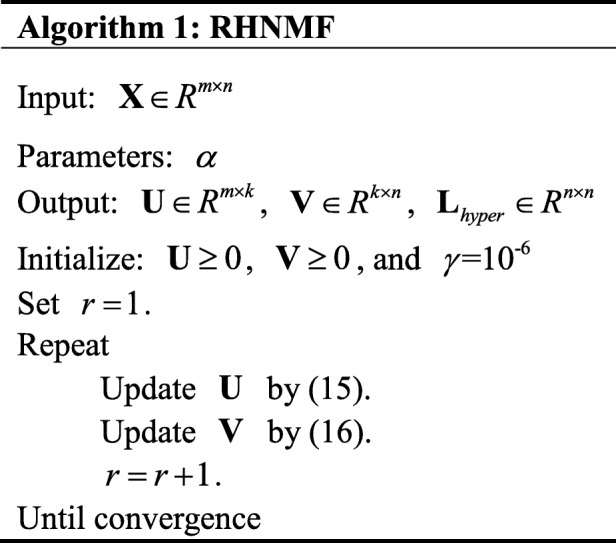


**Fig. 2:**
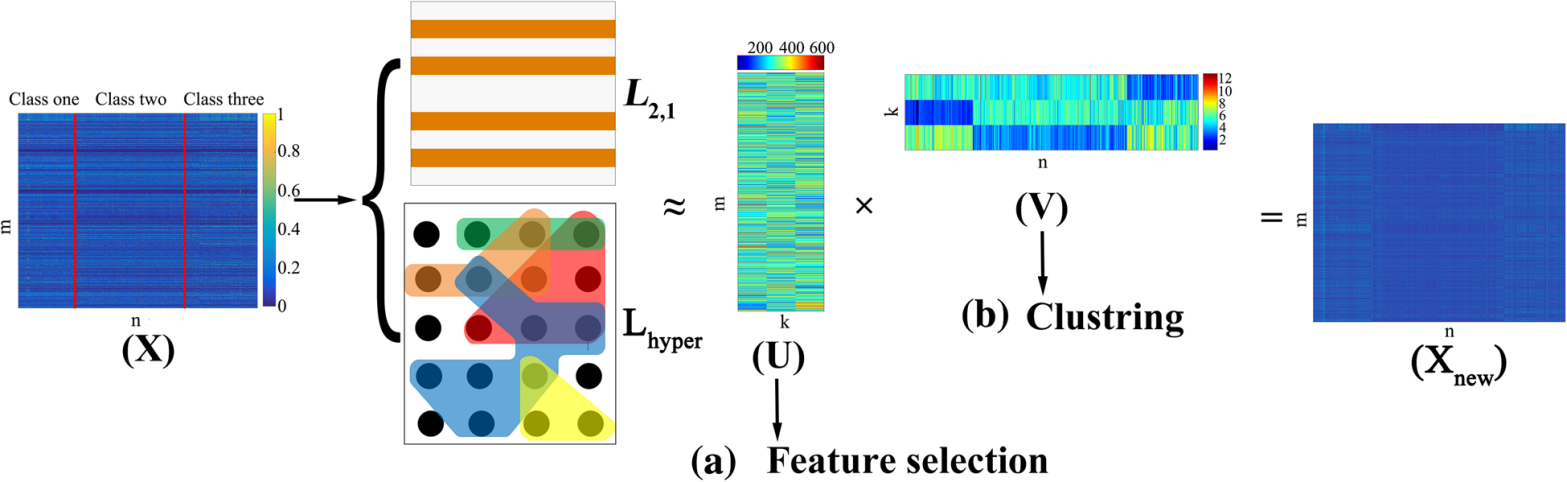
The whole framework of RHNMF

Finally, we use Fig. 2 to illustrate our model. From Fig. 2, we can see that the original data matrix consists of different types of data. The RHNMF method with *L*_2, 1_-norm constraint and hypergraph regularization has good robustness. We can perform feature selection on the basis matrix and perform sample clustering on the coefficient matrix.

### Convergence and complexity analysis

In this subsection, the computational costs of the RHNMF are presented. The general method to describe the computational complexity is to use arithmetic operations. Multiplicative iterative update rules guarantee **U** ≥ 0 and **V** ≥ 0. So we can iteratively update **U** and **V** until RHNMF’s objective function value is less than a sufficiently small number or the number of iterations exceeds the set maximum. It guarantees the convergence of the algorithm. Based on (15) and (16), we specifically analyze the arithmetic operations of each iteration of the RHNMF method. Assume that the original data matrix **X**^*m* × *n*^, *m* represents the number of genes, the number of samples is represented by *n*, *k* denotes the number of factors, *g* represents the number of nearest neighbors when constructing hyperedges in our algorithm. Therefore, we need 2*mnk* + 2(*m* + *n*)*k*^2^ + *n*(*g* + 3)*k* additions, 2*mnk* + 2(*m* + *n*)*k*^2^ + (*m* + *n*)*k* + *n*(*g* + 1)*k* multiplications, and (*m* + *n*)*k* divisions for (15) and (16). The overall costs of RHNMF method are *O*(*mnk*).

## Results and discussion

In this section, we apply the RHNMF model to cluster samples and select com-abnormal expression genes. To verify the validity of RHNMF, we compare it to other methods on multi-view dataset. These comparison methods include K-means, PCA, NMF [14], NMF*L*_2, 1_ [13], GNMF [11], HNMF [12], SHNMF [18], and RGNMF [19].

### Datasets

The Cancer Genome Atlas (TCGA) program applies high-throughput sequencing technology to understand the mechanisms of the occurrence and development of cancer cells [20]. In this experiment, we testify the performance of the RHNMF method in four multi-view datasets, including pancreatic cancer (PAAD_GE), head and neck squamous cell carcinoma (HNSC_GE), esophagus cancer (ESCA_GE), and cholangiocarcinoma (CHOL_GE). The datasets are downloaded from the TCGA. Any three of the four gene expression data are processed into multi-view datasets. Therefore, a total of four multi-view data have been formed. It is the gene expression data that after normal samples are removed are our data used in this paper. Table 1 lists the detailed information for the multi-view datasets.

**Table 1:** Summary of four multi-view datasets

Datasets	Samples	Genes	Classes	Views	Pv
PAAD_HNSC_CHOL_GE	610	20502	3	3	20502, 20502, 20502
PAAD_ESCA_CHOL_GE	395	20502	3	3	20502, 20502, 20502
PAAD_HNSC_ESCA_GE	757	20502	3	3	20502, 20502, 20502
HNSC_ESCA_CHOL_GE	617	20502	3	3	20502, 20502, 20502

Note: Datasets are different multi-view data. Classes represent the number of data categories (the type of cancer), views represent the number of data views (the type of cancer), and P_V_ represents the dimension of each view

### Parameter setting

**Fig. 3:**
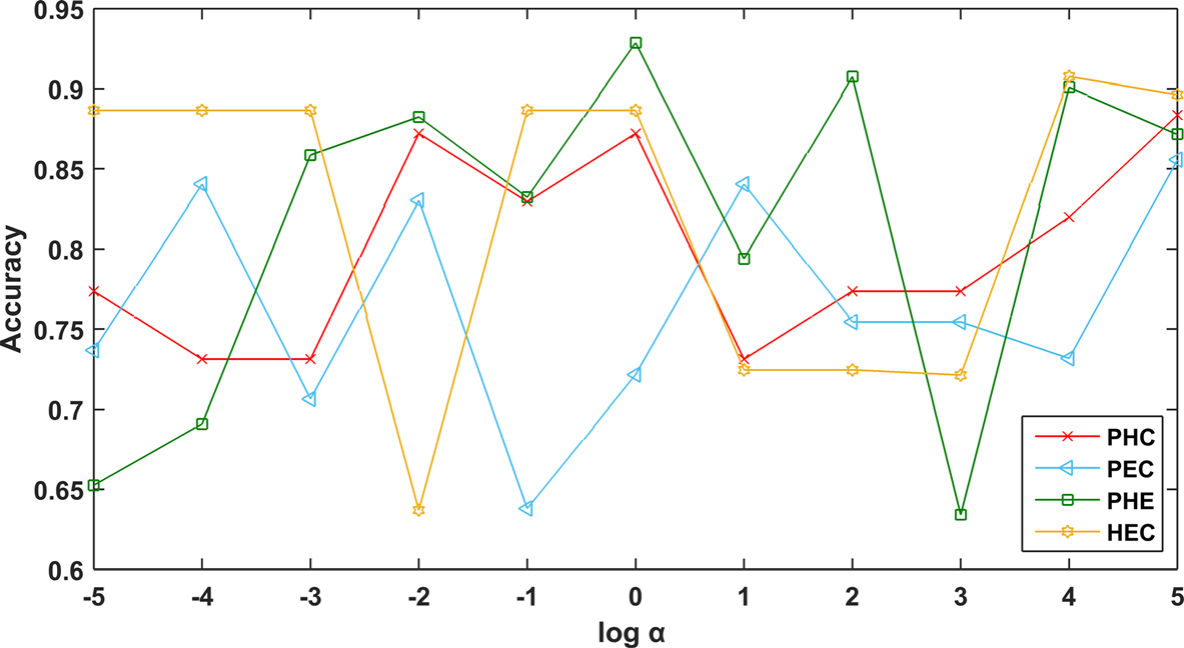
Performance of the RHNMF set with different values of *α*

In our proposed RHNMF method, the balance parameter *α* affects the experimental results. Because the value of the regularization parameter represents the degree of consideration of high-order relationship among data points, the value of the appropriate regularization parameter will contribute to the experimental results. So fivefold cross-validation is used to select the optimal parameters. The scope of the selection is {10^*r*^ : *r* ∈ {−5, −4, −3, …, 3, 4, 5}}. Figure 3 depicts the effect of parameter changes on RHNMF clustering performance. We can see from Fig. 3 that the hypergraph regularization parameters *α* are 10^5^, 10^5^, 10^0^, and 10^4^ on PAAD_HNSC_CHOL_GE, PAAD_ESCA_CHOL_GE, PAAD_HNSC_ESCA_GE, and HNSC_ESCA_CHOL_GE, respectively.

### Clustering results

In the experiment, we perform 50 times for each method. To illustrate the superiority of our algorithm, we compare it with other methods in the clustering of multi-view data. Then, we employ the K-means algorithm on the decomposed coefficient matrix for sample clustering.

#### Evaluation metrics

In the experiment, we employ two evaluation metrics to evaluate the clustering results [21, 22]. The first evaluation metric is accuracy (AC), which is the percentage of samples that are correctly clustered. The second evaluation metric is normalized mutual information (NMI), which indicates the similarity between the cluster set we obtained and the actual cluster set. Then, the AC is calculated by

17 $$ AC=\frac{\sum_{i=1}^n\delta \left({s}_i,\mathrm{map}\left({r}_i\right)\right)}{n}\times 100\%, $$

where *s*_*i*_ denotes the ground truth label and *r*_*i*_ represents the cluster label that is obtained in the clustering experiment. map(*r*_*i*_) denotes the mapping function that maps label *r*_*i*_ to the label *s*_*i*_ using the Kuhn–Munkres algorithm [23]. Then, *δ*(*x*, *y*) denotes a delta function. When *x* = *y*, *δ*(*x*, *y*) is 1; otherwise, *δ*(*x*, *y*) is 0. In addition, *n* represents the number of samples.

NMI represents the degree of similarity between two cluster sets and it has been widely used. For two cluster sets *C* and *C*^'^, NMI is expressed as:

18 $$ \mathrm{NMI}\left(C,{C}^{\hbox{'}}\right)=\frac{\mathrm{MI}\left(C,{C}^{\hbox{'}}\right)}{\max \left(H(C),H\left({C}^{\hbox{'}}\right)\right)}, $$

where *H*(*C*) and *H*(*C*^'^) represent the entropies of *C* and *C*^'^. MI(*C*, *C*^'^) represents the mutual information between two cluster sets.

#### Comparison of clustering performance

To illustrate the effectiveness of RHNMF, we perform the clustering experiment on the multi-view datasets. Then, we use AC and NMI to evaluate the clustering result. Finally, the details of clustering results are summarized in Table 2. According to Table 2, we can easily draw the following conclusions:

**Table 2:** Comparison of clustering performance in multi-view datasets

Datasets	PAAD_HNSC_CHOL_GE		PAAD_ESCA_CHOL_GE		PAAD_HNSC_ESCA_GE		HNSC_ESCA_CHOL_GE	
	AC (%)	NMI (%)	AC (%)	NMI (%)	AC (%)	NMI (%)	AC (%)	NMI (%)
K-means	57.19 ± 0.21	20.71 ± 0.74	52.24 ± 0.33	6.67 ± 0.48	46.79 ± 0.07	14.35 ± 0.30	54.62 ± 0.09	15.93 ± 0.10
PCA	57.71 ± 0.02	18.38 ± 0.32	47.02 ± 0.12	1.00 ± 0.01	46.98 ± 0.08	13.63 ± 0.32	48.95 ± 0.04	10.70 ± 0.06
NMF	48.28 ± 0.28	15.95 ± 0.08	52.56 ± 0.17	6.05 ± 0.15	46.41 ± 0.00	13.27 ± 0.02	48.87 ± 0.14	9.74 ± 0.09
GNMF	53.46 ± 0.24	17.23 ± 0.37	47.68 ± 0.01	1.52 ± 0.01	44.82 ± 0.10	14.18 ± 0.28	52.95 ± 0.09	15.29 ± 0.10
NMFL_2,1_	58.69 ± 0.00	26.19 ± 0.00	57.17 ± 0.09	21.58 ± 0.03	50.21 ± 0.14	22.38 ± 0.26	51.70 ± 0.18	15.62 ± 0.09
HNMF	65.70 ± 0.02	32.18 ± 0.19	51.36 ± 0.07	25.64 ± 0.02	64.63 ± 0.08	26.90 ± 0.15	58.63 ± 0.09	19.32 ± 0.05
SHNMF	66.40 ± 0.03	35.62 ± 0.31	52.10 ± 0.07	26.01 ± 0.01	63.85 ± 0.04	36.93 ± 0.01	58.96 ± 0.06	19.07 ± 0.04
RGNMF	79.33 ± 0.83	60.42 ± 0.19	75.44 ± 0.76	60.52 ± 0.69	79.98 ± 0.81	53.74 ± 1.25	72.49 ± 1.35	38.36 ± 1.17
RHNMF	*82.34 ± 0.71*	*62.26 ± 0.34*	*77.04 ± 0.65*	*63.96 ± 0.20*	*85.23 ± 0.62*	*60.05 ± 1.19*	*84.29 ± 0.98*	*52.72 ± 1.19*

Note: The best experimental results are highlighted in italics

(i) On these four multi-view datasets, the HNMF and SHNMF outperform the GNMF method, and the RHNMF also is higher than the RGNMF method. This is because the graph regularization only considers the intrinsic geometric relationships between pairs of samples. Hypergraph regularization and sparse hypergraph regularization, on the other hand, consider the manifold structure among more samples. That is, hypergraph Laplacian is able to find geometric information between multiple samples with similar embedding. This shows that the method of applying the hypergraph regularization term constraint has higher clustering accuracy.

(ii) According to whether there is *L*_2, 1_-norm constraints in the error function, we divide the seven methods based on NMF into three groups for comparison. The NMF*L*_2, 1_ is approximately 5% and 10% bigger than the NMF on AC and NMI, respectively. RGNMF exceeds GNMF by 27% and 41% on AC and NMI. RHNMF is higher than HNMF and SHNMF, by about 22% and 30% on the mean of AC and NMI. We can see that the methods with *L*_2, 1_-norm have better clustering results. This is because multi-view data has more samples, so there will be more noise and outliers. Fortunately, *L*_2, 1_-norm can enhance the robustness of the algorithm.

(iii) The NMF clustering results on the PAAD_ESCA_CHOL_GE and PAAD_HNSC_ESCA_GE datasets are not the worst. The reason may be that the improvement of traditional NMF will cause the loss of useful information and affect the clustering results. The clustering result of K-means is obtained by directly clustering the original data set without dimensionality reduction. From Table 2, we can see that its clustering results are acceptable because it considers all the information in the datasets without losing any information.

(iv) In Table 2, we can observe that our RHNMF method outperforms other methods. The clustering accuracy is increased by at least 5% and 6% on all datasets. Therefore, it is reasonable that the combination of the hypergraph structure and *L*_2, 1_-norm makes the clustering effect obviously.

The development of single-cell RNA sequencing (scRNA-seq) technology has enabled the measurement of gene expression in individual cells. This gives us an unprecedented opportunity to study biological mechanisms at the cellular level. The main single-cell analysis is to study the heterogeneity of cells, that is, to cluster a large number of cells into different groups. Therefore, in this subsection, we perform clustering experiments on single-cell datasets using the nine methods described above. The single-cell dataset for lung epithelial cells is available in the NCBI’s Gene Expression Omnibus (GEO GSE84147), including 540 cells (215 control, 275 idiopathic pulmonary fibrosis patients, and 50 interstitial lung disease patients) [24]. Table 3 lists the experimental results of the different methods on the lung epithelial cell dataset. Table 3 shows that the RHNMF method gives the best clustering performance. Specifically, our method’s AC and NMI are about 1% and 0.5% better than the second best result. The reason is that our method considers the robustness of the algorithm and the high-order relationship between the data. And this also shows that our approach applies not only to TCGA datasets but also to single-cell datasets.

**Table 3:** The clustering performance of the nine methods on single-cell dataset

Methods	K-means	PCA	NMF	GNMF	NMFL2,1	HNMF	SHNMF	RGNMF	RHNMF
AC (%)	76.16 ± 0.18	76.89 ± 0.64	77.19 ± 0.64	78.57 ± 0.47	78.15 ± 0.32	79.19 ± 0.26	78.36 ± 0.45	79.76 ± 0.13	*80.94 ± 0.07*
NMI (%)	38.29 ± 0.22	36.34 ± 0.77	38.27 ± 0.73	39.63 ± 0.53	41.05 ± 0.10	40.39 ± 0.26	39.12 ± 0.57	40.78 ± 0.04	*41.19 ± 0.03*

Note: The best experimental results are highlighted in italics

### Com-abnormal gene selection results

Cancer is the most common type of modern diseases, and it is a serious threat to human life and health. Changes in the genome often lead to cancer [25, 26]. Therefore, we select com-abnormal genes on the PAAD_ESCA_CHOL_GE dataset (to save space, we only list the experimental results on the PAAD_ESCA_CHOL_GE dataset.). From the consideration of the connection among multiple cancers, pancreatic cancer (PAAD), esophagus cancer (ESCA), and cholangiocarcinoma (CHOL) are studied.

In the experiment, the gene selection method used is introduced in [5]. We select 100 genes from each method for comparison. GeneGards (http://www.genecards.org/) can analyze the selected genes. GeneCards is a searchable comprehensive database that succinctly provides genomes, proteomics, and all known and predicted human genes. Tables 4, 5, and 6 list the detailed experimental results.

**Table 4:** Performance comparison of com-abnormal gene selection in multi-view datasets

Methods	*N*	Com-abnormal genes
PCA	25	**KRT19**, SPINK1, PRSS1, MUC6, VIM, HLA-A, SERPINA1, **CTSB**, KRT8, **GNAS**, ANXA2, HSPB1, HLA-C, **KRT5**, **S100A6**, PKM, HSP90AA1, ENO1, KRT17, MALAT1, **COL1A1**, ALDOA, LIPF, **TMSB10**, RPLP0
NMF	15	**KRT19**, SPINK1, PRSS1, HLA-A, SERPINA1, **CTSB**, KRT8, SPP1, **GNAS**, **KRT5**, **S100A6**, SERPINA3, **COL1A1**, **TMSB10**, RPLP0
GNMF	24	**KRT19**, PRSS1, MUC6, VIM, HLA-A, SERPINA1, **CTSB**, KRT8, **GNAS**, ANXA2, HSPB1, HLA-C, **KRT5**, **S100A6**, PKM, HSP90AA1, ENO1, KRT17, MALAT1, **COL1A1**, ALDOA, LIPF, **TMSB10**, RPLP0
NMF*L*_2, 1_	31	CEACAM5, **KRT19**, VIM, HLA-A, SERPINA1, **CTSB**, KRT8, CEACAM6, **GNAS**, ANXA2, HSPB1, HLA-C, **KRT5**, LAMC2, **S100A6**, ITGB1, PKM, HSP90AA1, ENO1, KRT17, MALAT1, MMP11, ITGB4, **COL1A1**, HSPG2, ALDOA, LDHA, LGALS3BP, S100A11, **TMSB10**, RPLP0
HNMF	32	CEACAM5, **KRT19**, VIM, HLA-A, SERPINA1, **CTSB**, KRT8, CEACAM6, **GNAS**, ANXA2, HSPB1, HLA-C, **KRT5**, **S100A6**, ITGB1, PKM, HSP90AA1, ENO1, S100A9, KRT17, LCN2, MALAT1, ITGB4, **COL1A1**, HSPG2, ALDOA, HSP90B1, LDHA, LGALS3BP, S100A11, **TMSB10**, RPLP0
SHNMF	31	CEACAM5, **KRT19**, VIM, HLA-A, SERPINA1, **CTSB**, KRT8, CEACAM6, **GNAS**, ANXA2, HSPB1, HLA-C, **KRT5**, **S100A6**, ITGB1, PKM, HSP90AA1, ENO1, S100A9, KRT17, LCN2, MALAT1, **COL1A1**, HSPG2, ALDOA, HSP90B1, LDHA, LGALS3BP, S100A11, **TMSB10**, RPLP0
RGNMF	33	EGFR, CCND1, **KRT19**, CD44, PRSS1, VIM, SLC2A1, **CTSB**, **GNAS**, ANXA2, HSPB1, HLA-C, **KRT5**, LAMC2, **S100A6**, ITGB1, PKM, HSP90AA1, ENO1, S100A9, H19, KRT17, ANXA1, MALAT1, ITGB4, **COL1A1**, ALDOA, HSPA1A, TNC, LDHA, LGALS3BP, S100A11, **TMSB10**
RHNMF	34	CEACAM5, **KRT19**, VIM, HLA-A, SERPINA1, **CTSB**, KRT8, CEACAM6, SPP1, **GNAS**, ANXA2, HSPB1, HLA-C, **KRT5**, KRT18, **S100A6**, ITGB1, PKM, HSP90AA1, ENO1, KRT17, HSPA5, LCN2, MALAT1, ITGB4, **COL1A1**, HSPG2, ALDOA, HSP90B1, LDHA, LGALS3BP, S100A11, **TMSB10**, RPLP0

Note: Bold genes denote that they are selected simultaneously by these eight methods. Underlined genes denote that they can be selected by RHNMF. *N* represents the number of com-abnormal genes selected for every method

**Table 5:** Detailed analysis of the com-abnormal genes selected only by the RHNMF method

Gene ID	Gene ED	Related GO annotations	Related diseases	Relevance score
3875	KRT18	Poly(A) RNA binding and scaffold protein binding	Cirrhosis, cryptogenic, and nonalcoholic steatohepatitis	11.99, 11.76, 2.61
3309	HSPA5	Calcium ion binding and ubiquitin protein ligase binding	Borna disease and Wolfram syndrome	11.46, 9.13, 0.88

**Table 6:** Summary of the same com-abnormal genes discovered by eight methods

Gene ID	Gene ED	Related GO annotations	Related diseases	Relevance score
3880	KRT19	Structural molecule activity and structural constituent of cytoskeleton	Lung cancer and thyroid cancer	31.72, 24.50, 20.66
1508	CTSB	Peptidase activity and cysteine-type peptidase activity	Keratolytic winter erythema and occlusion of gallbladder	24.10, 10.61, 1.22
2778	GNAS	GTP binding and signal transducer activity	McCune-Albright syndrome, somatic, mosaic, and pseudohypoparathyroidism Ia	28.52, 9.69, 1.78
3852	KRT5	Structural molecule activity and scaffold protein binding	Epidermolysis bullosa simplex, Dowling-Meara type and epidermolysis bullosa simplex, Weber-Cockayne type	24.03, 13.77, 0.17
6277	S100A6	Calcium ion binding and calcium-dependent protein binding	Endometrial cancer and pancreatic cancer	19.09, 6.61, 1.44
1277	COL1A1	Identical protein binding and platelet-derived growth factor binding	Caffey disease and osteogenesis imperfecta, type I	8.85, 19.64, 1.22
9168	TMSB10	Actin binding and actin monomer binding	Actin binding and actin monomer binding	3.31, 1.29, 1.22

In Table 4, the *N* is obtained by matching the differential genes selected by each method to the virulence gene pool of every integrated dataset. The RHNMF method gives the largest *N*. This is because *L*_2, 1_-norm is not sensitive to noise and outliers. And the row sparsity produced by the *L*_2, 1_-norm constraint will contribute to the selection of com-abnormal genes. Therefore, our method is effective for the selection of com-abnormal genes.

The com-abnormal expressed genes selected by RHNMF and not selected by other methods are listed in Table 5. The relevant score refers to the correlation between genes and diseases. The higher relevance score means that abnormal expression of the gene is more likely to cause malignant tumor production. And we can see that the relevance scores of KRT18 with PAAD, ESCA, and CHOL are 11.99, 11.76, and 2.61, respectively. KRT18 (Keratin 18) is a protein-coding gene. It encodes the type I intermediate filament chain keratin 18. KRT18 has been shown to be associated with the appearance of PAAD, ESCA, and CHOL [27–29]. HSPA5 probably plays a role in facilitating the assembly of multimeric protein complexes inside the endoplasmic reticulum. The relevance scores of HSPA5 with PAAD, ESCA, and CHOL are 11.46, 9.13, and 0.88, respectively. HSPA5 has to do with the occurrence of PAAD, ESCA, and CHOL [30–32]. This suggests that biologists need to further study KRT18 and HSPA5 to better understand the link among PAAD, ESCA, and CHOL. And it shows that the RHNMF method is useful in selecting the com-abnormal genes.

Table 6 lists the same com-abnormal genes discovered by these eight methods. Table 6 is similar to Table 5. As we all know, a gene may be linked to a variety of diseases, and the emergence of a disease is the result of multiple genes acting together. KRT19 has the highest correlation score in these three diseases. Together with KRT8, it helps to link the contractile apparatus to dystrophin at the costameres of striated muscle. KRT19’s related diseases are lung cancer and thyroid cancer. Some literature has shown that KRT19 is related to PAAD, ESCA, and CHOL [33–35]. The com-abnormal genes in Table 6 are found in all eight methods, implying the importance of these genes. The com-abnormal gene selection by considering the links among different cancers is of great significance to PAAD, ESCA, and CHOL research.

## Conclusions

In this paper, we design a novel non-negative matrix factorization model called RHNMF for sample clustering and the selection of com-abnormal genes. On the one hand, considering the low-dimensional manifold information existing in the high-dimensional data, the hypergraph regularization term is applied to the objective function of RHNMF. On the other hand, we use *L*_2, 1_-norm on the error function to enhance the robustness of the algorithm. Experimental results on the multi-view datasets demonstrate the superiority of the RHNMF in comparison with other representative methods.

However, the proposed method inevitably has limitations. For example, our method uses a traditional hypergraph, which does not capture high-order discriminant manifold information. For future work, we will introduce sparse hypergraph to solve the above problem.

## Data Availability

All data in this paper is available in https://cancergenome.nih.gov/.

## Abbreviations

AC: Accuracy
CHOL_GE: Cholangiocarcinoma
Convex-NMF: Convex non-negative matrix factorization
ESCA_GE: Esophagus cancer
GNMF: Graph regularized non-negative matrix factorization
HNMF: Hypergraph regularized non-negative matrix factorization
HNSC_GE: Head and neck squamous cell carcinoma
NLCF: Non-negative local coordinate factorization
NMF: Non-negative matrix decomposition
NMF*L*_2, 1_: Robust non-negative matrix factorization
NMFs: Sparse non-negative matrix factorizations
NMI: Normalized mutual information
PAAD_GE: Pancreatic cancer
RGNMF: Robust graph regularized non-negative matrix factorization
RHNMF: Robust hypergraph regularized non-negative matrix factorization
Semi-NMF: Semi-non-negative matrix factorization
TCGA: The Cancer Genome Atlas
